# Complete genome sequence of *Planococcus koreensis* isolated from soil in Fort Collins, Colorado

**DOI:** 10.1128/mra.00193-25

**Published:** 2025-04-17

**Authors:** Paige Gruber, Ashley Freedman, Kendall Malmstrom, Bradley R. Borlee, Carolina Mehaffy

**Affiliations:** 1 Department of Microbiology, Immunology and Pathology, Colorado State University 3447, Fort Collins, Colorado, USA; DOE Joint Genome Institute, Berkeley, California, USA

**Keywords:** Nanopore, *Planococcus korensis*, genome

## Abstract

The complete genome of *Planococcus koreensis* was obtained using Nanopore MinION sequencing after isolation from soil in Colorado. The assembled genome contains one circular contig with 3,519,105 bp, 3,606 genes, 419 pseudogenes, and 47.62% guanine-cytosine content. This discovery provides a fully assembled *P. koreensis* genome available at the National Center for Biotechnology Information.

## ANNOUNCEMENT

Whole-genome sequencing was performed as part of a Course-based Undergraduate Research Experience (CURE) at the Department of Microbiology, Immunology, and Pathology, Colorado State University. On 27 August 2024, an orange-pigmented bacteria isolate was obtained from a soil sample taken near Dixon Reservoir, Fort Collins, CO, at coordinates 40°33′12″ N 105°8′25″ W, at 5,200 ft elevation. After plating on tryptic soy agar for 24–48 h at room temperature, the isolate was streaked for isolation and identified as *Planococcus koreensis* via matrix-assisted laser desorption/ionization - time of flight (score = 1.74) (Bruker). *Planococcus* spp. produced a variety of metabolites with potential utility for bioremediation and industrial applications (1–3).

The isolate stained gram variable, as confirmed by previous literature (4). The isolate was grown in LB for 48 h before genomic DNA (gDNA) isolation using the Monarch Genomic DNA Purification Kit (#T3010S). Given the isolate’s gram variable status, G+ and G− extraction protocols were performed. Library preparation from each gDNA preparation was performed using the Oxford Nanopore Technologies Rapid PCR Barcoding Kit 24 v.14 (SQK-RPB114.24) using the manufacturer’s protocol and 25 PCR cycles. gDNA was not size-selected. Quality was checked via agarose gel electrophoresis and Qubit (dsDNA HS Assay Kit [Q32851]). Sequencing was performed with Oxford Nanopore Technologies MinION Flow Cell R10 Version (FLO-MIN114) using MinKnow v.24.06.8, Basecalling Fast Model v.4.3.0, 400 bp, and Min Q score of 8. Barcode trimming was performed post-run in MinKnow v.24.06.8 with default parameters.

Assembly and annotation were performed using Galaxy Server v.24.1.4.dev0 (5–7). Raw reads were pre-processed using Necat v.0.0.1_update20200803 + galaxy0 (8) in UseGalaxy.eu with default parameters and an estimated genome size of 3.5 Mb. All other tools were run on UseGalaxy.org. Concatenate Multiple Data Sets v.0.2 (9) was used to concatenate the corrected reads, followed by assembly with Flye v.2.9.5 + galaxy0 (10, 11), revealing a circular genome with one contig of 3,537,709 bp and coverage of 53%. Quast v.5.2.0 + galaxy1 (12–15) was run and indicated the contig had an N50 = 3,537,709. The assembly was polished using Racon v.1.5.0+galaxy1 (16), resulting in a polished genome of 3,519,105 bp. Annotation was performed using Prokka v.5.2.0 + galaxy1 (17, 18) with kingdom: bacteria. The isolate was confirmed as *Planococcus koreensis* by comparing the 16S rDNA gene from the annotated genome against the 16S rDNA bacteria and archaea database using BLAST (19) (99.87% identity and 97% query cover).

**Fig 1:**
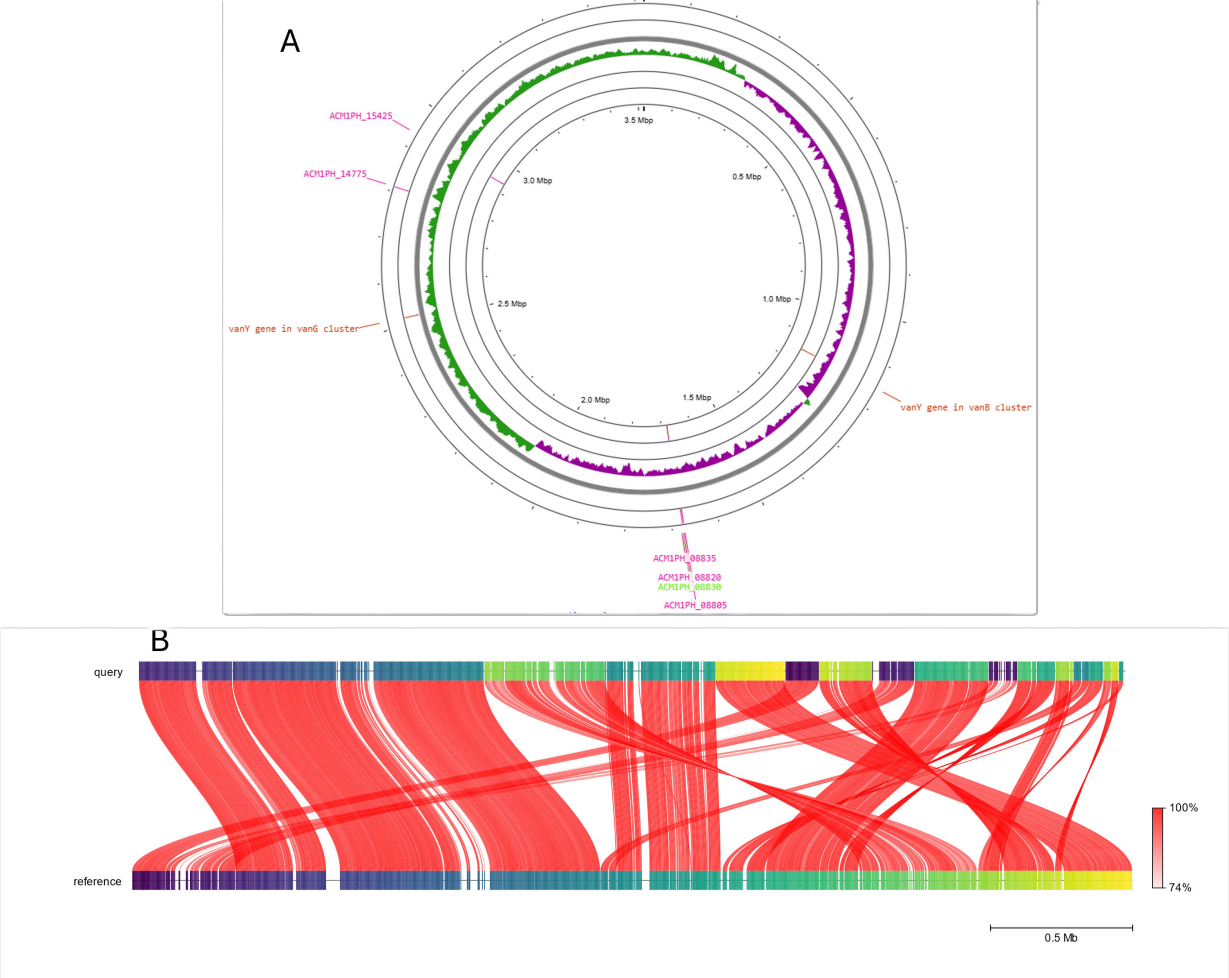
(A) *P. koreensis*, Fort Collins isolate, complete genome. Features show two predicted glycopeptide resistance genes (vanY genes), five genes, and one pseudogene predicted to be involved in carotenoid biosynthesis. Guanine-cytosine (GC) skew is shown (GC skew, purple; GC skew+, green). (B) Results from FastANI (average nucleotide identity [ANI]) against the *P. koreensis* reference genome (accession number JACHHE01) resulting in an ANI of 97.1598%. Each red line segment denotes a reciprocal mapping between the query (top) and reference (bottom) genomes. Shade of red represents the ANI percentage. Colors on the query and reference genome (horizontal segments) represent orthologous fragments of 3,000 bp.

The complete genome was visualized in Proksee (20) using the consensus fasta file (Fig. 1). FastANI v.1.1.0 (21) was used to compare the isolate genome with the reference, incomplete, 22-contig genome available at the National Center for Biotechnology Information (NCBI), *Planococcus koreensis* (accession number JACHHE01), resulting in 97.1598% average nucleotide identity with 1,061 orthologous matches out of 1,144 query sequence fragments (Fig. 1). The genome of *Planococcus koreensis* presented in this report is 3,519,105 bp, with a guanine-cytosine content of 47.62%, assembled in one circular contig (N50 = 3,519,105 bp). CARD analysis (https://card.mcmaster.ca/analyze/rgi) (22) identified two genes associated with glycopeptide resistance. Upon submission to the NCBI, the genome was annotated using the NCBI Prokaryotic Genome Annotation Pipeline (23–25), resulting in 3,606 coding sequences and 419 pseudogenes, including 5 genes and 1 pseudogene predicted to be carotenoid biosynthetic genes (Fig. 1).

## Data Availability

This whole-genome assembly has been deposited at GenBank with accession number ASM4779632v1 under biosample SAMN46743435. The annotated genome can be accessed under accession number CP181055.1. The version described in this paper is the first version. Raw reads were submitted to the National Center for Biotechnology Information Sequence Read Archive with accession number SRR32300276.
